# Focal fibrocartilaginous dysplasia in the thoracic vertebra: A case report

**DOI:** 10.3892/ol.2014.2331

**Published:** 2014-07-09

**Authors:** BO SUN, ZHI HONG ZHANG, XUAN YING CHEN, SHAN HU HUANG, ZHI LI LIU

**Affiliations:** 1 Department of Orthopaedics, The First Affiliated Hospital of Hainan Medical College, Haikou, Hainan 570102, P.R. China; 2 Department of Orthopaedics, The First Affiliated Hospital of Nanchang University, Nanchang, Jiangxi 330006, P.R. China

**Keywords:** focal fibrocartilaginous dysplasia, thoracic vertebra, rib

## Abstract

Focal fibrocartilaginous dysplasia (FFCD) is a rare, paraneoplastic disease that often presents in children and teenagers. Previous studies have reported cases of lesions in the proximal tibia and distal femur, as well as lesions in the upper extremities. The present study describes a case of FFCD on the transverse process and the rib. The imaging findings were found to correspond with the typical observations of FFCD and a biopsy from the nidus revealed pathological results similar to those of previous reports. Thus, the present study demonstrated that FFCD affects tubular bones as well as flat bones. Further studies are required to investigate the underlying mechanism and treatment of FFCD.

## Introduction

Focal fibrocartilaginous dysplasia (FFCD) is a rare, paraneoplastic disease that presents in children and teenagers. Less than 100 cases have been reported in the English literature since the original description by Bell *et al* (1). Previous studies have reported cases of lesions in the proximal tibia and distal femur, as well as lesions in the upper extremities. However, to the best of our knowledge, there are no reports of FFCD on the transverse process or the rib. Thus, the present study is the first report of a case of FFCD in the vertebra and the rib. The aim of this report is to testify that FFCD may occur in tubular bones and flat bones.

## Case report

A 38-year-old male (body mass index, 21.01) presented at The First Affiliated Hospital of Nanchang University (Nanchang, China) with pneumonia and lesions on the left first thoracic vertebra, which were detected using chest computed tomography (CT). The patient reported that he had experienced a tolerable level of pain in the area three times during the previous ten years, with no history of trauma. No other family members had suffered from a similar condition. The initial consultation was performed on 28/05/2013. Informed consent was obtained from the patient.

**Figure 1:**
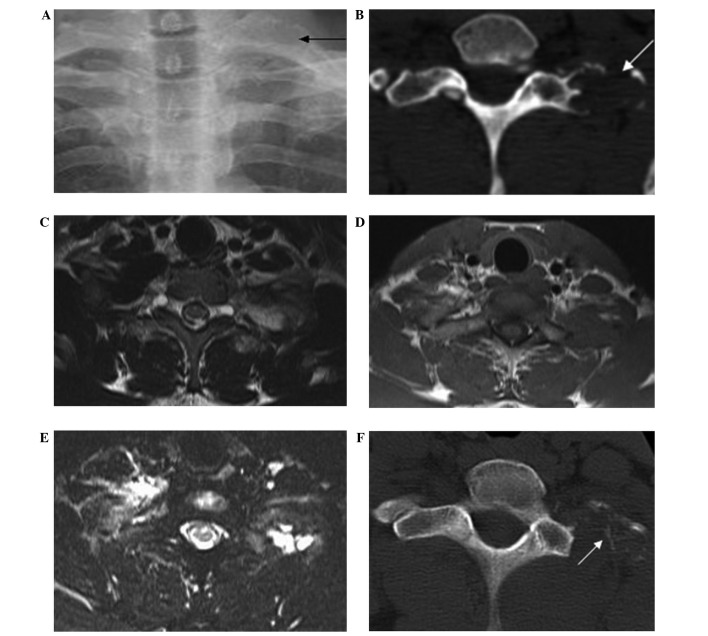
X-ray, computed tomography (CT) and magnetic resonance imaging (MRI) of the lesion. (A) X-ray revealing a lucent and inflated lesion in the left first transverse process and the costal head (black arrow). (B) Chest CT showing that the two sides of the lesion were sclerosing and that the cortical bone of the lesion was thinning, but intact. No signs of periosteal reaction or cortical destruction were observed. (C–E) MRI showing (C) a low signal of the lesion in the T1-weighted image, (D) an intermediate signal of the lesion in the T2-weighted images and (E) a high signal of the lesion in the fat suppression images. (F) Follow-up chest CT after two months exhibits a patchy high-density shadow in the primary lesion (white arrow). The cortical plate was incomplete and swelling was apparent in the surrounding soft tissue.

Physical examinations revealed that the patient was healthy and ambulated without difficulty. The patient exhibited a full range of motion of the neck and upper extremities. Furthermore, no masses were palpable. X-ray (Fig. 1A) and chest CT scans (Fig. 1B) were performed and revealed a well-defined, unilateral, lucent and inflated lesion in the left first transverse process and the costal head (estimated size, 2.2×2.4 cm). The two sides of the lesion, which connected with the normal bony tissue, were sclerosing. In addition, the cortical bone of the lesion was found to be thinning; however, there was no periosteal reaction or cortical destruction. Magnetic resonance imaging (MRI) demonstrated a low signal of the lesion in the T1-weighted image (TE 10.0 and TR 581.0; Fig. 1C) and an intermediate signal in the T2-weighted images (TE 130.0 and TR 4,710.0; Fig. 1D). A high signal was observed in the lesion in the fat suppression images (TE 130.0 and TR 6,380.0; Fig. 1E). Thus, the radiologists proposed that it may be an aneurysmal bone cyst. A full 99mTC-MDP scan showed that there was no sign of malignant transformation in the patient’s skeletal system, and the results from the laboratory examinations and the tumor markers, including α-fetoprotein, carcinoembryonic antigen and cancer antigen 19-9, were all within normal limits.

**Figure 2:**
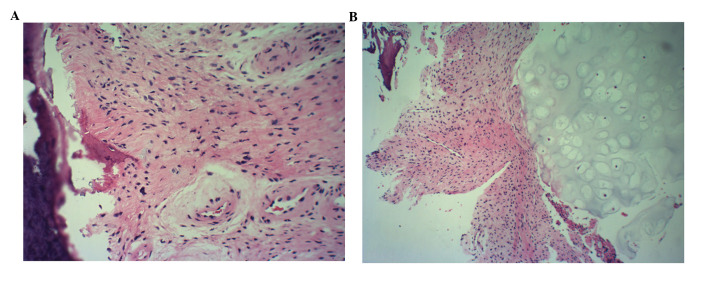
Microscopic observations of the lesion. (A) Irregularly-shaped bony trabeculae surrounded by numerous fibroblasts. Hematoxylin and eosin (H&E) stain; magnification, ×200. (B) Cell stroma composed of relatively uniform spindle cells and cartilaginous components without significant cellular atypia. H&E stain; magnification, ×100.

Curettage of the focal lesion and an incisional biopsy were performed due to the possibility of the lesion being an aneurysmal bone cyst. The surgery lasted 120 min and the estimated blood loss was 200 ml. A frozen section obtained during surgery did not provide a specific diagnosis. After one week, the pathologic results from a specimen of the lesion (Fig. 2) demonstrated irregularly-shaped bony trabeculae surrounded by numerous fibroblasts (Fig. 2A), which were detected using hematoxylin and eosin (H&E) staining (magnification, ×200). Furthermore, the cell stroma was observed to be composed of relatively uniform spindle cells and cartilaginous components without significant cellular atypia (H&E; magnification, ×100; Fig. 2B). The patient was informed of the findings and, as the patient only exhibited slight back pain, no further treatment was administered and the patient was closely observed.

Two months after surgery, the patient was followed up. Chest CT (Fig. 1F) demonstrated a patchy, high-density shadow in the primary lesion. The patient is currently pain-free and is not prevented from performing any activities. The patient will be followed up annually.

## Discussion

FFCD is an uncommon, benign bone lesion that deforms the long bones in children. FFCD predominantly occurs in the proximal tibia and rarely occurs in the long bones of the upper limbs. To the best of our knowledge, the present study is the first study to report a case of FFCD on the transverse process of the rib. Furthermore, the patients reported in previous studies have all been adolescents or infants, with only Ohno *et al* (2) and Hermann *et al* (3) reporting cases of patients aged >25 years; the current study presents the case of an adult patient. FFCD lesions in the lower limbs usually present in patients of a younger age (1,4–6), while upper limb involvement usually occurs in older individuals (6,7). The present study reported the case of a 38-year-old male, thus, FFCD lesions in the vertebrae or ribs may be more likely to present in post-adolescent or middle-aged individuals. However, further investigations are required to assess this hypothesis.

The lower extremities are the most frequent site for FFCD; however, its pathogenesis is controversial. For example, Bell *et al* (1) proposed that in lesions of the tibia, failure of the mesenchymal anlage to differentiate in the area of the pes anserinus, as well as the persistence of a focus of fibrocartilage, may impair growth on the medial aspect of the proximal tibia. Jouve *et al* (8) hypothesized that FFCD is a pathology of the pes anserinus insertion, which interferes with its physiological migration during growth through creating a pseudo-epiphysiodesis. Langenskiöld (9) proposed that trauma during delivery may be a predisposing factor by causing necrosis of the medial aspect of the physis. However, the pathogenesis of lesions in the transverse process or the rib remains unclear.

FFCD may be easily diagnosed using imaging techniques. The typical radiographic signs include a well-defined, lucent defect in the metaphyseal cortex of the medial long bone and sclerosis along the lateral border of the lesion (10). MRI may be performed in cases where the diagnosis is uncertain. Furthermore, MRI excludes the possibility of soft tissue lesions. The typical appearance of FFCD on T1- and T2-weighted MRI slices consists of a low-signal area (corresponding to the radiolucent area) and an intermediate signal (corresponding to the sclerotic area) (11). In the present case, the chest CT and X-ray images showed a well-defined, unilateral, lucent and inflated lesion in the left first transverse process and the costal head. The cortex was thinning, but intact and there were no signs of periosteal reaction or cortical destruction. In the present case, the radiographic and MRI findings corresponded with the typical features of FFCD. However, a specific diagnosis was not determined, as FFCD had not been reported in the vertebrae or ribs in previous studies.

FFCD is a variant of fibrous dysplasia (FD). Radiologically, FFCD is similar to conventional FD, in that the lesion is well demarcated and exhibits cortical expansion, but with an intact cortex. In order to diagnose FFCD it must be differentiated from dyschondrosteosis, Ollier’s disease, neurofibromatosis and trauma (12). In contrast to FFCD, FD lesions are purely fibrous and show no evidence of cartilaginous or osseous elements (4). In the present case, the most important differential diagnosis was osteofibrous dysplasia, as typical osteofibrous dysplasia exhibits a similar image morphology to FFCD, with frosted glass-like lesions, inflated and thickened lesions in the bone, cortical plate thinning and no periosteal reaction or cortical destruction. However, osteofibrous dysplasia always present as a palpable mass with bone deformities. Furthermore, a number of cases present with pathologic fractures and high alkali phosphatase levels (13). Additional differential diagnoses include osteoblastic sarcoma and aneurysmal bone cysts.

It has been hypothesized that each of the deformities associated with FFCD may spontaneously correct or progressively improve and studies have reported an incidence of spontaneous correction ≤45% (5). Through observing the healing process of FFCD, Jouve *et al* (8) demonstrated that an infantile active growth plate of the proximal tibia is able to correct a varus deformity of ≤30°. Peroneal nerve palsy (12,14) and overcorrection to valgus deformity were reported to be a consequence of certain cases that were treated with an osteotomy (15). Surgical treatment may be avoided if there is no evidence of the deformity increasing or pathological fracture at presentation; however, in the present case, the lesion was potentially an aneurysmal bone cyst and, although the tumor was small, the patient requested that it was removed, thus, it was excised.

In lesions in any of the four limbs, FFCD is consistently associated with angular deformities in children. The associated deformities commonly result in limb-length inequalities, with disparities of ≤30 mm (1,4,9). Furthermore, a limb-length discrepancy of 7.7 cm has previously been reported in the upper limbs (6). The present study reported a case of FFCD of the vertebra and rib. Surgery was performed to treat the patient and a long-term follow-up was recommended in order to assess the likelihood of FFCD recurrence.

In conclusion, the present case proposes that FFCD may affect tubular bones as well as flat bones. However, the underlying mechanism and treatment of FFCD requires further investigation.
